# A Novel Strategy to Promote Equity and Access in Interventional Cardiology: Early Insights From the 2024-2025 ACC Clinical Trial Research Cohort

**DOI:** 10.1016/j.jscai.2025.103923

**Published:** 2025-11-11

**Authors:** Priscilla Wessly, Giorgio A. Medranda, Katelyn Bruno, Aderonke Adeniyi, Zainab Atiyah Dakhil, Hussein AbuDaya, Johanna Contreras, Diana Otero, Olufunso Odunukan, Joji Varghese

**Affiliations:** a Aurora Sinai/Aurora St. Luke’s Medical Center, Aurora Cardiovascular and Thoracic Services, Milwaukee, Wisconsin; b Division of Cardiology, Department of Medicine, NYU Langone Hospital – Long Island, Mineola, New York; c Division of Cardiovascular Medicine, Department of Medicine, University of Florida, Gainesville, Florida; d Department of Cardiovascular Medicine, Wake Forest School of Medicine, Winston-Salem, North Carolina; e Ibn Al Bitar Cardiac Centre, University of Baghdad, Baghdad, Iraq; f Division of Cardiovascular Medicine, Department of Medicine, University of Alabama at Birmingham, Birmingham, Alabama; g Mount Sinai Hospital, New York, New York; h Division of Cardiovascular Medicine, Department of Medicine, University of California San Diego, La Jolla, California; i Dignity Health, Yavapai Regional Medical Center, Prescott, Arizona; j HeartCare Texas, North Richland Hills, Texas

**Keywords:** collaboration, diversity, health equity, mentorship, research engagement

## Abstract

**Background:**

The American College of Cardiology (ACC) launched the Clinical Trials Research (CTR) program in 2019 to increase representation of historically underrepresented early-career investigators in cardiovascular research. In 2024, ACC introduced REACH (Research, Equity, and Access for Cardiac Health), a new subcohort within CTR focused on addressing disparities in structural heart disease research and recruitment. This analysis aimed to evaluate the diversity of the 2024-2025 CTR and REACH cohorts and assesses the program’s short-term impact on research engagement, mentorship, and professional development.

**Methods:**

This was a cross-sectional voluntary survey of 22 items offered to all participants following completion of the program in March 2025. Responses were collected anonymously to ensure confidentiality, with data encrypted during transit and stored securely.

**Results:**

Among the 2024-2025 ACC CTR cohort, 48 of 51 participants (94.1%) completed the survey, with equal sex distribution (50% women, 50% men) and 25% identifying as Hispanic. Reported short-term outcomes included 52% gaining new mentorship outside their institution, 23% receiving speaking invitations to national meetings, 81% forming new collaborations, and 79% identifying new research opportunities. In the ACC CTR REACH cohort, 60% reported new external mentorship, 40% received national speaking invitations, 60% formed collaborations, and 88% accessed at least 1 new research opportunity.

**Conclusions:**

The 2024-2025 ACC CTR and REACH programs fostered mentorship, collaboration, and research engagement among early-career investigators while advancing diversity in sex and ethnicity.

## Introduction

Although cardiovascular medicine has advanced significantly, care delivery continues to be marked by persistent disparities across different populations. These disparities stem from a complex interplay of factors, including race, ethnicity, sex, socioeconomic status, and health care access.^1^, ^2^, ^3^, ^4^, ^5^, ^6^ The cardiovascular workforce continues to lack the diversity necessary to deliver equitable, high-quality care, further perpetuating these imbalances.^7^^,^^8^ Addressing such disparities requires a multifaceted approach encompassing medical education, training, research, clinical practice, and policy reform. In response, the American College of Cardiology (ACC) launched the Clinical Trials Research (CTR) program in 2019 to increase the number of historically underrepresented leaders in cardiovascular research.^9^^,^^10^ The program targets a diverse pool of early-career to mid-career clinicians and researchers, offering a structured curriculum aligned with core clinical research competencies, professional development opportunities, and a capstone project.^10^ In 2024, the ACC expanded this initiative with the launch of project REACH (Research, Equity, and Access for Cardiac Health), a specialized CTR subcohort composed of multidisciplinary participants including researchers, imaging cardiologists, interventional cardiologists, and cardiothoracic surgeons, with a focus on structural heart disease (SHD).^10^ Through a year-long collaborative curriculum, participants receive advanced training, mentorship, and project development support to identify and address disparities in SHD trial design, access, and enrollment.^10^ This analysis aimed to describe the demographic characteristics and professional diversity of the 2024-2025 CTR and REACH cohorts and to assess the program’s short-term impact on research engagement, mentorship access, and perceived professional development.

## Materials and methods

### Survey design

The 2024-2025 ACC CTR program, including both the ACC CTR and CTR REACH cohorts, started on June 20, 2024, and finished on February 28, 2025. This was a cross-sectional survey offered to all participants to complete voluntarily immediately following completion of the program in March 2025. This online survey included 22-items designed to detail participants’ backgrounds (demographic characteristics, professional profile, and research experience) and highlight short-term outcomes from the program (new mentorships, speaker invitations, new collaborations, and new research opportunities). A copy of the survey viewed by participants can be found in the Supplemental Appendix S1. Responses were collected electronically.

### Data collection

Responses were collected anonymously to ensure confidentiality, with data encrypted during transit and stored securely. The online platform used enabled export to Microsoft Excel for initial data management. The dataset was structured to allow comparisons with future ACC CTR cohorts to evaluate longitudinal trends in diversity and research engagement over time.

### Ethical considerations

This survey-based evaluation did not include protected health information and was therefore deemed exempt from institutional review board approval. Participants provided implied consent by voluntarily completing the survey, and anonymity was ensured throughout the data collection and analysis process.

### Statistical analysis

Descriptive statistics summarized participant demographic characteristics, professional characteristics, and program outcomes using Excel version 16.95 (Microsoft Corp). Categorical variables (eg, sex, ethnicity, race, and institution type) were reported as frequencies and percentages, while continuous variables (eg, years in cardiology practice) were analyzed using medians and IQRs due to nonnormal distribution. Percentages were calculated based on available responses. For the REACH cohort subset, outcomes were summarized descriptively using frequencies and percentages, without additional hypothesis testing, given the exploratory focus and smaller sample size. Graphs were created using Prism version 10.4.2 (GraphPad Software).

## Results

### 2024-2025 ACC CTR cohort demographic characteristics

**Figure 1:**
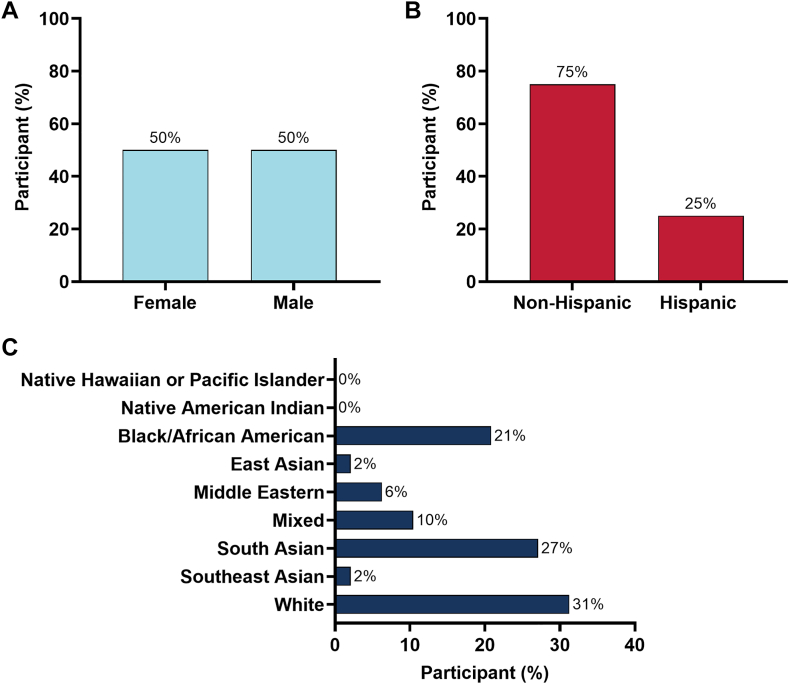
**Demographic characteristics of 2024-2025 ACC CTR Cohort.** Broken down by (**A**) sex, (**B**) ethnicity, and (**C**) race.

The 2024-2025 ACC CTR program contained 51 participants, of whom 48 completed the survey (94.1%). Among those who completed the survey in the ACC CTR cohort, the majority were aged 31 to 45 years (n = 37 of 48) and a minority were aged 46 to 55 years (n = 11) (Figure 1, Central Illustration). Participants who completed the survey were 50% women (n = 24) and 50% men (n = 24). With regard to ethnicity, 25% were Hispanic (n = 12), and 75% were non-Hispanic (n = 36). With regard to race, 2.1% (n = 1) were East Asian, 27.1% (n = 13) South Asian, 2.1% (n = 1) Southeast Asian, 20.8% (n = 10) Black, 31.3% (n = 15) White, 10.4% (n = 5) of mixed race, none Native American Indian or Alaskan Natives, 6.3% (n = 3) were Middle Eastern, and none Native Hawaiian or Pacific Islanders (Figure 1, Central Illustration).

**Central Illustration:**
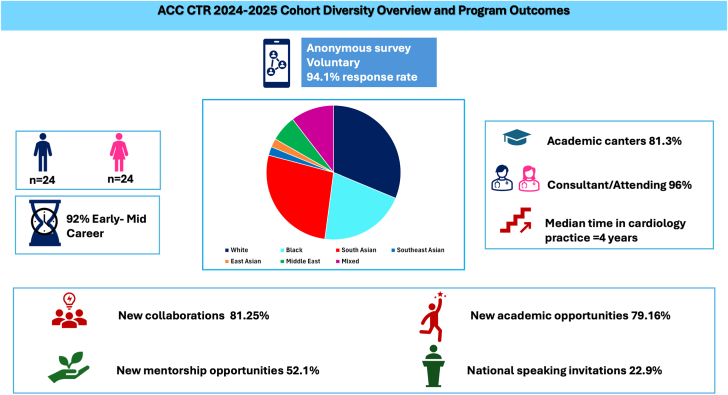
**2024-2025 ACC CTR cohort diversity overview and program short-term outcomes.** ACC, American College of Cardiology; CTR, clinical trials research.

### 2024-2025 ACC CTR cohort professional experience

**Figure 2:**
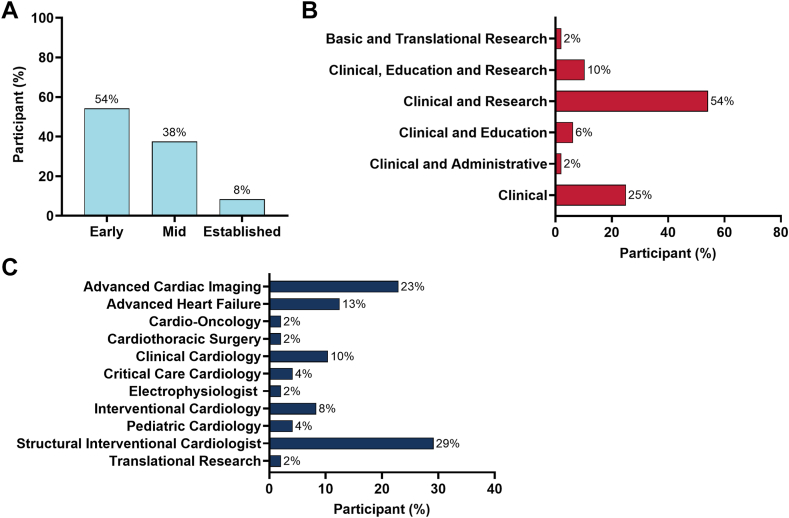
**Professional experience of 2024-2025 ACC CTR cohort.** The ACC CTR cohort consists of (**A**) years of experience (early career: <5 years; mid-career: 5-15 years; and established career: >15 years), (**B**) career path, and (**C**) specialty.

Among those who completed the survey, 54.2% (n = 26) were at early-career phase (<5 years from training), 37.5% (n = 18) were at mid-career level (5-15 years from training), and the remaining 8.3% (n = 4) had established career (Figure 2, Central Illustration). The majority of participants who completed the survey identified as having both a clinical and research career path (54.2%, n = 26) (Figure 2). Approximately 95.8% (n = 46) were clinical cardiologists or subspecialty clinical cardiologists, 2.1% (n = 1) were cardiothoracic surgeons and 2.1% (n = 1) were doctors of philosophy (Figure 2). Most participants who completed the survey were affiliated with a teaching hospital (91.7%, n = 44) and an academic center (81.3%, n = 39) (Figure 2, Central Illustration). With regard to research effort, 8.3% (n = 4) reported no dedicated research time, 14.6% (n = 7) reported 1% to 9%, 50.0% (n = 24) reported 10% to 20%, 6.3% (n = 3) reported 30% to 44%, 14.6% (n = 7) reported 45% to 60%, and 6.3% (n = 3) reported 75% to 100% research effort (Figure 2). Most participants (85.4%, n = 41) had experience in clinical trials, serving in roles such as subinvestigator (n = 27), site principal investigator (n = 21), or co-principal investigator (n = 11) (Figure 2). Familiarity with artificial intelligence (AI) was reported by 64.6% (n = 31), and 25.0% (n = 12) had used AI tools in research settings.

### 2024-2025 ACC CTR cohort program outcomes

**Figure 3:**
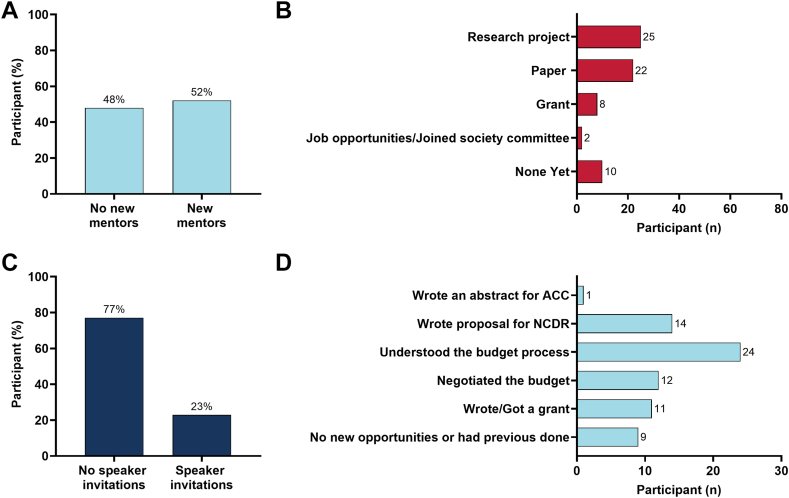
**Short-term outcomes from the 2024 to 2025 ACC CTR and REACH cohorts.** (**A**) The ACC CTR cohort received speaker invitations, found new mentors, obtained new research opportunities, and developed new collaborations. (**B**) The REACH cohort received speaker invitations, found new mentors, obtained new research opportunities, and developed new collaborations. (**C**) Overall cohort new research opportunity. (**D**) Overall cohort new collaboration.

At completion of the 2024-2025 ACC CTR program, participants who completed the survey reported that 52.1% (n = 25) found new mentorship outside their institution, and 22.9% (n = 11) received new speaking invitations to national meetings (Figure 3, Central Illustration). Additionally, 81.25% (n = 39) had formed new collaborations, 79.16 % (n = 38) had reported new research opportunities (writing an abstract for ACC Scientific Sessions, writing a proposal for the National Cardiovascular Data Registry [NCDR], writing a grant, negotiating a budget, and/or developing a better understanding of the budget process) (Figure 3, Central Illustration).

### 2024-2025 ACC CTR REACH cohort

The 2024-2025 ACC CTR REACH cohort contained 27 participants, of whom 25 completed the survey (92.6%). Among those who complete the survey in the ACC CTR REACH cohort, 28.0% were women (n = 7) and 72.0% were men (n = 21). Familiarity with AI was reported by 68.0% (n = 17), with 28.0% (n = 7) having used AI in research before the program. After completion of the ACC CTR REACH program, participants who completed the survey reported that 60.0% (n = 15) found new mentorship outside their institution and 40.0% (n = 10) received speaking invitations to national meetings. Additionally, 60.0% (n = 15) formed new collaborations, and 88.0% (n = 22) received at least 1 new research opportunity (writing an abstract for ACC Scientific Sessions, writing a proposal for the NCDR, writing a grant, negotiating a budget, and/or developing a better understanding of the budget process).

## Discussion

The principal findings of this cross-sectional survey of the 2024-2025 ACC CTR and CTR REACH cohorts are as follows: (1) The program achieved sex parity, with 50% of CTR participants identifying as women, and included representation across multiple racial and ethnic groups, including White, Black/African American, Hispanic, and South Asian participants; (2) among CTR respondents, 52% established new mentorships outside their home institutions, 23% received speaking invitations to national conferences, 85% initiated new collaborations, and 81% reported at least 1 new career opportunity related to their participation; (3) In the REACH cohort, 60% reported new external mentorships, 40% received speaking invitations, 60% initiated new collaborations, and 88% identified at least 1 new career opportunity associated with their participation in the program.

### Building a diverse research pipeline

The 2024-2025 ACC CTR cohort demonstrates meaningful progress toward its mission of fostering a diverse and inclusive early-career research workforce. Despite a growing number of women in cardiovascular medicine, historical data show that women have represented <16% of first authors and only 11% of steering committee members in heart failure trials, with no significant improvement over time.^11^ Systemic barriers, such as limited mentorship, restricted advancement opportunities, and the lack of a structured pipeline for diverse research leadership, continue to perpetuate this gap.^11^^,^^12^ By achieving sex parity, the program addresses the longstanding underrepresentation of women in cardiology clinical trials—a persistent obstacle to equitable trial design and leadership. The ACC CTR’s intentional and strategic approach marks an essential step toward lasting change, helping open doors for more women to lead trials, serve on steering committees, and gain national visibility through research and speaking opportunities. The inclusion of 28% women in the REACH cohort, given the historical underrepresentation of women in procedural subspecialties (8.2% in interventional cardiology), underscores the program’s success in addressing sex disparities in SHD trials.

Similarly, despite increasing diversity at the entry level of medical training, persistent underrepresentation of racial and ethnic minorities in research leadership roles remains as demonstrated: only 1.4% of principal investigators and 6.9% of active physicians are Black/African American (13.5% of the general population), and only 3.9% principal investigators and 5.9% of physicians are Hispanic (18.6% of the general population).^7^ The inclusive approach of the ACC CTR program—with deliberate outreach to underrepresented racial and ethnic groups—represents a critical intervention in correcting this imbalance. The cohort’s racial and ethnic diversity is equally notable, including White, Black/African American, Hispanic, South Asian, Southeast Asian, East Asian, Middle Eastern, and multiracial participants. The program also made a commendable effort to include international cardiologists from Iraq, Brazil, and Portugal in the training initiative.

### Practice setting and leadership development

With >50% of participants in their first 0 to 5 years of independent practice and 27% within 5 to 10 years, the ACC CTR program effectively engages early-career and mid-career professionals, positioning them for long-term contributions to clinical research. Its inclusive design spans 11 cardiology subspecialties—including structural interventional cardiology, advanced cardiac imaging, heart failure, cardio-oncology, pediatric cardiology, cardiothoracic surgery, and translational research—underscoring a commitment to reducing disparities across specialized fields. Participants represent diverse practice settings, with most from urban academic centers and meaningful representation from community hospitals and private practices with academic ties. This geographic and institutional diversity fosters culturally competent trial leadership and promotes equitable recruitment strategies. The program’s structured exposure to senior clinical researchers facilitates mentorship, collaboration, and career development, especially vital for those in nonacademic settings. While the current ACC CTR and REACH subcohort focused on individuals who have completed formal training, extending similar pipeline programs to trainees, could further amplify efforts to promote health equity by engaging underrepresented groups earlier in their careers. Future cohorts and program development could explore the feasibility and impact of such initiatives.

### Immediate impact

The ACC CTR program yielded significant academic gains, highlighting its immediate impact on research capacity and career advancement. Among those who completed the survey, an impressive 85.4% reported new collaborations—ranging from manuscript writing and joint grant proposals to ACC abstract submissions—while 81.3% experienced at least 1 new professional opportunity, such as negotiating a study budget, preparing an NCDR proposal, or submitting work to a national meeting. Notably, 22.9% received new speaking invitations to national conferences, opening doors that have historically been less accessible to diverse investigators. For many, these were first-time experiences, emphasizing the program’s effectiveness in breaking down barriers for underrepresented groups. These outcomes contribute to a more inclusive culture in cardiovascular research and have lasting implications for mentorship, institutional change, and the training of future generations.

### AI in cardiovascular research

AI increasingly influences cardiovascular research and clinical decision making, and the ACC CTR program addressed this trend through targeted training. Before the program, 64.6% of participants were familiar with AI, and only 25% had used it in research. Sessions on clinical research design, AI integration in practice, and AI tools for enhancing care and trials were highly valued. AI can promote diversity in trial recruitment by enabling automated, unbiased screening of electronic health records to identify underrepresented patients. Tools like natural language processing and geospatial algorithms help extract clinical data and target diverse communities, while remote consent and monitoring improve accessibility for rural and underserved populations. AI supports more inclusive and representative clinical trials by streamlining data and enabling real-time recruitment adjustments. By incorporating AI into the curriculum, the program equips this cohort to lead the future of cardiovascular research, promoting innovation, enhancing clinical impact, and advancing diversity in trial design and patient recruitment. Long-term follow up will be required to understand further the impact introducing AI has had on participants of the program.

### Patient advocacy and community engagement

Patient-centered trial design was a distinctive component of the cohort’s training, with in-person sessions emphasizing the importance of humanizing the research experience. Hearing directly from patients and patient advocates about their motivations and barriers to participation was described as “eye-opening,” reinforcing the ethical imperative of inclusivity in clinical research. Discussions also underscored the value of community engagement, particularly in underserved areas, where partnerships with community leaders and culturally aligned organizations can build trust, improve recruitment, and support trial retention. By fostering these connections, the program helps ensure that clinical trials reflect real-world populations, address disparities in cardiovascular research, and enhance patient representation.

### Policy and systemic change

The 2024-2025 ACC CTR cohort marks a critical step toward systemic reform in cardiovascular research by demonstrating how intentional, policy-aligned diversity efforts can reshape the clinical trials landscape. With 50% women participation and robust racial and subspecialty representation, the program directly responds to longstanding calls—such as those in the 2020 AHA/ACC Consensus Conference—for a research workforce that mirrors the patient population.^13^ This shift is not symbolic; it enables more inclusive trial design, improved recruitment of underrepresented populations, and results that are more applicable to real-world care. By integrating targeted training initiatives like REACH, leveraging AI for equitable recruitment, and fostering community partnerships, the CTR program sets a practical precedent for institutions and policymakers committed to closing equity gaps. Its outcomes reflect not just individual advancement, but a replicable model for structural change in clinical research.

### Limitations

This cross-sectional survey has several limitations. The small sample size of 48 participants, including 25 in the REACH cohort, limits statistical power and generalizability. Its cross-sectional design and reliance on self-reported data restrict causal inferences and introduce potential response bias, although anonymity likely mitigated some of this risk. Additionally, the cohort had limited representation from rural and community hospitals—a gap that should inform future recruitment strategies. Finally, longitudinal tracking of alumni outcomes, such as publications, grant funding, and leadership roles in clinical trials, will be essential to fully assess the program’s long-term impact on the research workforce. While the immediate postprogram survey provided valuable insights, it did not specifically assess changes in participants’ familiarity with or usage of AI in research. Future studies should incorporate longer-term follow up assessments to evaluate the sustained impact of the ACC CTR and REACH programs on participants’ integration of AI into cardiovascular research.

## Conclusion

The 2024-2025 ACC CTR and REACH programs represent meaningful shifts toward a more inclusive and forward-thinking research workforce. The program continues to cultivate a pipeline of diverse leaders prepared to drive equity in cardiovascular and SHD clinical research. The immediate professional gains highlight its effectiveness in breaking down systemic barriers in hopes of bridging the longstanding gap in recruiting underrepresented patient populations, ultimately leading to more inclusive trials, stronger scientific validity, and improved health outcomes across diverse communities.
