# Supplementary material for: A Novel Strategy to Promote Equity and Access in Interventional Cardiology: Early Insights From the 2024-2025 ACC Clinical Trial Research Cohort
Source: J Soc Cardiovasc Angiogr Interv. 2025 Nov 11;5(3 Suppl):103923. doi: 10.1016/j.jscai.2025.103923 (PMC13112825; doi:10.1016/j.jscai.2025.103923)
Supplement: Supplemental Material [file mmc1.pdf]

Diversity and Insights of ACC Clinical Trials Research Program (CTR) Cohorts

This survey is intended for candidates who were recruited in the ACC CTR Program.

It will take less than two minutes to answer the survey.

We appreciate your time and insights with this.

1. What is your age?

- ◯ <30
- ◯ 31-45
- ◯ 46-55
- ◯ >55

2. What is your sex?

- ◯ Male
- ◯ Female

3. What is your ethnic background?

- ◯ Hispanic / Latino
- ◯ Non-Hispanic / Latino
- ◯ Other (please specify)

4. What race do you identify yourself?

- ◯ East Asian (Chinese, Japanese, Korean)
- ◯ South Asian (Indian, Pakistani, Bangladesh)
- ◯ Southeast Asian (Filipino, Vietnamese, Thai)
- ◯ Black or African American
- ◯ White
- ◯ Mixed
- ◯ Native American Indian or Alaska Native
- ◯ Middle Eastern or North African
- ◯ Native Hawaiian or Pacific Islander

5. What is your CTR cohort year entry?

6. Which of the following best describes your specialty?

- ◯ Clinical cardiology
- ◯ Cardiac imaging
- ◯ Interventional cardiology
- ◯ Electrophysiology
- ◯ Heart failure
- ◯ Clinical research / physician scientist
- ◯ Allied health professional - clinical pharmacist, nurse, nurse practitioner, radiologist, etc.
- ◯ Other (please specify)

7. Which of the following best describes your current role / designation?

- ◯ Cardiology fellow / in training
- ◯ Cardiology consultant / attending
- ◯ Non-cardiology fellow / in training
- ◯ Non-cardiology consultant / attending
- ◯ Allied health professional - clinical pharmacist, nurse, nurse practitioner, radiologist, etc.

- ○ Other (please specify)

8. Time in cardiology practice since finishing training in cardiology?

- ○ I am still in training
- ○ <5 years
- ○ 5-10 years
- ○ 10-15 years
- ○ >15 years

9. What type is your institution?

- ○ Urban
- ○ Rural
- ○ Suburban

10. Is your institute a teaching / academic hospital?

- ○ Yes
- ○ No

11. What is the type of your main institution?

- ◯ Academic center
- ◯ Non-academic
- ◯ Academic affiliate
- ◯ Private practice with academic affiliation
- ◯ Private practice without academic affiliation
- ◯ Federal rural
- ◯ Federal urban
- ◯ VA
- ◯ Other (please specify)

12. What mostly describes your career path?

- ☐ Clinical
- ☐ Clinical / research
- ☐ Education
- ☐ Basic research
- ☐ Translation
- ☐ Other (please specify)

13. From where did you hear FIRST about ACC CTR?

- ◯ ACC newsletters / website
- ◯ A CTR alumni
- ◯ A CTR faculty
- ◯ Friend
- ◯ Social media
- ◯ Other (please specify)

14. Do you have any prior participation in clinical trials?

- ◯ Yes
- ◯ No

15. If the above answer is yes, what was your role in the clinical trial?

- [ ] National principal investigator
- [ ] Site principal investigator
- [ ] Principal investigator of site NIH grant
- [ ] Principal investigator of site grant
- [ ] Co-principal investigator
- [ ] Sub-investigator
- [ ] Steering committee
- [ ] DSMB
- [ ] Not applicable

16. Were you familiar with Artificial Intelligence (AI) in cardiovascular research prior to the program?

- ◯ Yes
- ◯ No

17. Did you use certain AI software in research prior to the program?

◯ Yes

◯ No

18. Did you find mentorship from outside your institution as a result of the ACC CTR program?

◯ Yes

◯ No

19. What percentage of your time is dedicated to research?

20. Have you received any speaking invitations to a national meeting or another institution as a result of this program?

◯ Yes

◯ No

21. As a result of the program, have you

formed new collaborations for:

- [ ] Research project
- [ ] Writing a paper
- [ ] A grant
- [ ] Other (please specify)

22. During the program, was this the first time that you:

- [ ] Wrote an abstract for ACC (or any other meeting)
- [ ] Wrote a grant
- [ ] Wrote a proposal for NCDR4
- [ ] Understood the budget process
- [ ] Negotiated a budget
- [ ] Other (please specify)

Done

Powered by

SurveyMonkey®

See how easy it is to create surveys and forms.

Privacy & Cookie Notice
